# Common Host Responses in Murine Aerosol Models of Infection Caused by Highly Virulent Gram-Negative Bacteria from the Genera Burkholderia, Francisella and Yersinia

**DOI:** 10.3390/pathogens8040159

**Published:** 2019-09-21

**Authors:** Graeme C. Clark, Angela Essex-Lopresti, Karen A. Moore, E. Diane Williamson, Roman Lukaszewski, Konrad Paszkiewicz, Jonathan David

**Affiliations:** 1 Chemical, Biological and Radiological Division, DSTL Porton Down, Salisbury SP4 0JQ, UK; 2 Biosciences, University of Exeter, Geoffrey Pope Building, Stocker Road, Exeter EX4 4QD, UK

**Keywords:** RNAseq, pulmonary, infection, melioidosis, tularemia, plague, respiratory

## Abstract

Highly virulent bacterial pathogens cause acute infections which are exceptionally difficult to treat with conventional antibiotic therapies alone. Understanding the chain of events that are triggered during an infection of a host has the potential to lead to new therapeutic strategies. For the first time, the transcriptomic responses within the lungs of Balb/C mice have been compared during an acute infection with the intracellular pathogens *Burkholderia pseudomallei*, *Francisella tularensis* and *Yersinia pestis*. Temporal changes were determined using RNAseq and a bioinformatics pipeline; expression of protein was also studied from the same sample. Collectively it was found that early transcriptomic responses within the infected host were associated with the (a) slowing down of critical cellular functions, (b) production of circulatory system components, (c) lung tissue integrity, and (d) intracellular regulatory processes. One common molecule was identified, Errfi1 (ErbB receptor feedback inhibitor 1); upregulated in response to all three pathogens and a potential novel marker of acute infection. Based upon the pro-inflammatory responses observed, we sought to synchronise each infection and report that 24 h p.i. of *B. pseudomallei* infection closely aligned with 48 h p.i. of infection with *F. tularensis* and *Y. pestis*. Post-transcriptional modulation of RANTES expression occurred across all pathogens, suggesting that these infections directly or indirectly modulate cell trafficking through chemokine expression/detection. Collectively, this unbiased NGS approach has provided an in-depth characterisation of the host transcriptome following infection with these highly virulent pathogens ultimately aiding in the development of host-directed therapies as adjuncts or alternatives to antibiotic treatment.

## 1. Introduction

Understanding the biological responses that are triggered by Gram-negative pathogens during the course of infection within a host marks a critical step in the development of new and improved therapies [1,2]. In addition, for highly virulent pathogens, improving our knowledge as to the various strategies used to modulate the host response that permit colonisation, replication and dissemination during the manifestation of the disease is essential for identifying approaches required in order to yield new/novel host-directed targets for therapeutic intervention [3]. 

The innate immune system acts as the first line of defence against invading pathogens via pattern recognition receptors (e.g., Toll-like, NOD-like families) for PAMPs, leading to the recruitment of immune cells to the site of infection and a cascade of pro-inflammatory responses including cytokine and chemokine release [4]. However, highly pathogenic organisms are adept at subverting or evading the host immune response, particularly during the early phases of infection [4,5,6]. Typically, the diseases caused by highly virulent Gram-negative species from the genera *Burkholderia*, *Francisella* and *Yersinia* have an intracellular phase to their life cycle and have also been identified as having immunomodulatory properties leading to the late onset of symptoms, delayed diagnoses and ultimately increased mortality rates. Some of these properties account for their classification as Tier 1 or 2 Biothreat Agents by the Centre for Disease Control. 

The Gram-negative bacterium *Burkholderia pseudomallei*, endemic to Southeast Asia and Northern Australia, causes the potentially fatal disease melioidosis and is a common cause of community acquired septicemia within these locations [7,8]. Natural human infections occur percutaneously through skin abrasions and by the inhalation of aerosols from ground water. The disease exists in both acute and chronic forms, ranging from a localised infection to septicemia involving multiple organs, most commonly the spleen, liver and lung within humans and experimental mouse models [9,10,11,12]. The disease has the ability to become latent and reappears several years later possibly at the onset of an immunosuppressive condition such as late-onset diabetes.

*Yersinia pestis* is the causative agent of the severe zoonotic disease known as plague. Endemic within rodent populations, plague crosses over into the human population causing cases of human disease commonly in South-Western USA, South America, Central and Southern Africa, Madagascar, India, Russia and China. Dependent on the route of exposure, plague can take one of three forms: Bubonic, septicemic or pneumonic [13,14]. Of these, pneumonic plague arising either by direct infection via the respiratory route, or as a secondary infection from untreated bubonic plague, is an acute lethal disease [15,16,17]. Seasonal outbreaks occur regularly in Madagascar and the 2017 outbreak was one of the most serious with a predominance of pneumonic plague and ~9% case fatality rate [18].

*Francisella tularensis* is the causative agent responsible for the disease tularemia which is endemic in Scandinavia and North America [19]. Whilst several forms of disease occur naturally, pneumonic tularemia is the most lethal and has 30–60% mortality if left untreated [20]. *F. tularensis* has also been described as a “stealth pathogen” being able to evade arms of the immune response [21,22]. Previous transcriptomics studies have identified global alterations in the host response to the bacteria using rationally designed chip-based approaches which have provided some insight into the process of pulmonary infection [23,24]. 

Previously each organism, along with the diseases that they cause, has been studied separately and typically through the analysis of clinically relevant samples (e.g., blood, broncho-alveolar fluid). The purpose of this study was to investigate commonalities in the host response to each pathogen, in order to identify common points of intervention in pathogenesis and to assess the feasibility of developing novel broad-spectrum therapeutic strategies. The acute nature of the diseases that they cause mean that comparison of the host responses induced by each Biothreat Agent, rather than other lung pathogens, across a clinically relevant time window of between 72–96 h was most appropriate. To do this we have taken established murine (Balb/C) models of each disease which authentically represent the clinical syndromes in order to fully characterise the events occurring at the primary foci of infection of the lung following lethal inhalational challenges with either *B. pseudomallei* K96543, *F. tularensis* SchuS4 or *Y. pestis* CO92. 

Firstly, we have mapped the acute response of the lung with time to aerosolised pathogen using unbiased RNA sequencing (RNAseq) in order to elucidate the host response and the impact of immunomodulatory properties of these organisms on cytokines production during infection. Secondly, using parallel, statistically-powered animal studies we have for the first time sought commonalities in the host response that are triggered during infection with each bacterium. 

## 2. Results

### 2.1. Acute Pulmonary Infection of Balb/C Mice with Highly Virulent Bacterial Pathogens

Balb/C mice were challenged by the aerosol route with multiples of the lethal dose of either *F. tularensis* SchuS4, *B. pseudomallei* K96253 or *Y. pestis* CO92. It was found that *B. pseudomallei* colonised and replicated in the lung at a faster rate than either *Y. pestis* or *F. tularensis* over the first 24 h p.i. (Figure S1). From 48 h p.i. there was a general increase in bacterial numbers with time for all three pathogens. Coupled with these observations was an associated increase in the severity of clinical signs and lung weights (i.e., highlighting changes in organ integrity and/or oedema) during disease progression; only *F. tularensis* infection did not lead to statistically significant increases in lung wet weights (Figure S2). 

### 2.2. Innate Immune Responses During B. pseudomallei Infection 

**Figure 1:**
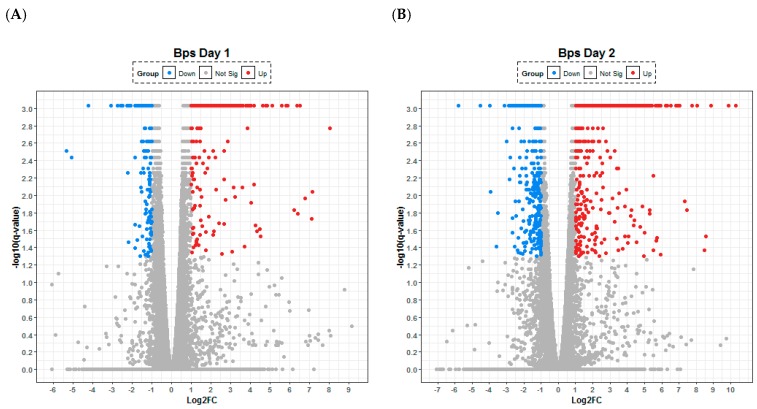

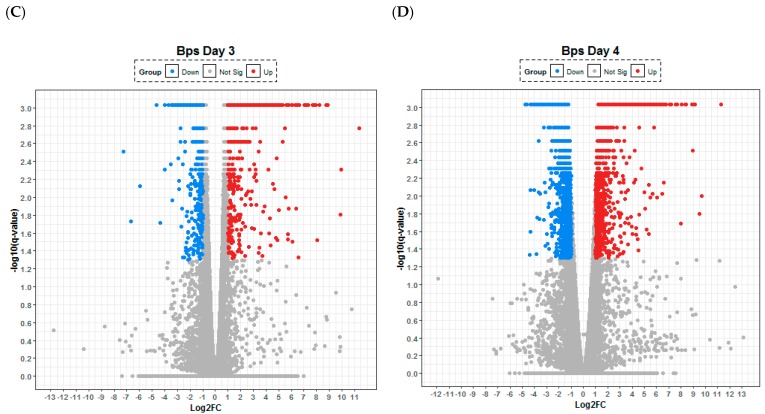
Lung transcriptome following aerosol infection of Balb/C mice with *Burkholderia pseudomallei*. Temporal changes are presented after (**A**) 24, (**B**) 48, (**C**) 72 and (**D**) 96 h following infection with the organism. Any gene that has log-fold change less than 2 versus uninfected control are labelled in grey, significantly up- (red) and down-regulated (blue). The statistical significance (−log10 of the *p*-value) of each gene within the transcriptome is shown on the *y*-axis.

**Figure 2:**
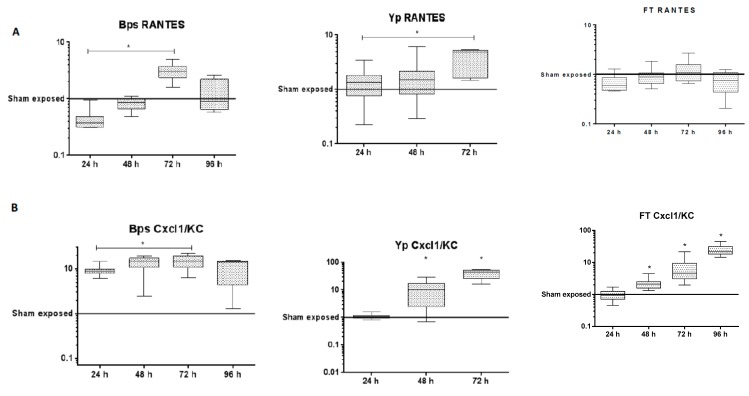
Concentrations of (**A**) RANTES and (**B**) CXCL1/KC within lung tissue following exposure to *B. pseudomallei*, *Y. pestis* or *F. tularensis*. Statistical significance was determined by the Kruskall–Wallis test with Dunn’s multiple comparisons test (* *p* < 0.05).

The transcriptional response to *B. pseudomallei* lung infection was observed to be dominated by the upregulation of genes involved in the innate immune response (Table 1). A consistent picture was observed across the first 48 h p.i. with the majority of the upregulated transcripts relating to the production of chemokines (CXCL2, 6, 9 and 10) involved in cellular recruitment and of cytokines (IL-6, IL-17) involved in acute inflammation; which can be visualised by volcano plots as a relative small, defined set of high upregulated and downregulated transcripts (Figure 1). It is noteworthy that from 24 to 72 h p.i., the transcript IRG1 for the immune response gene 1 (negative regulator of TLR-mediated responses) was upregulated the most, perhaps reflecting active modulation of the host response by the organism. Coupled to this finding was the downregulation of the regulatory molecule microRNA-467 which has been associated with a variety of acute viral infections and is thought to have a role in apoptosis, which in turn may support the development of chronic infections. The lung cytokine profiles generated by Luminex array within this study broadly agreed with previously published data [11] with interleukins-6, -12 and -17 all upregulated both within the transcriptome and the proteome from 48 h p.i. (Table S1). Consistent with analysis of the transcriptome, the secretion of molecules involved in cell recruitment/attraction/stimulation such as CXCL1 (kalikrein, KC; a chemokine required for neutrophil attraction) was significantly upregulated through time (Figure 2). In contrast with this trend was the consistent early downregulation of RANTES (CCL5; a chemotactic cytokine for T cell, eosinophils and basophils) within the proteome (Figure 2); but with subsequent significant increases in RANTES at 72 h p.i within both the transcriptome and proteome.

### 2.3. Absence of IFN-γ and the Upregulation of Elastases Indicate Active Modulation of the Host Lung Responses by Yersinia pestis 

**Figure 3:**
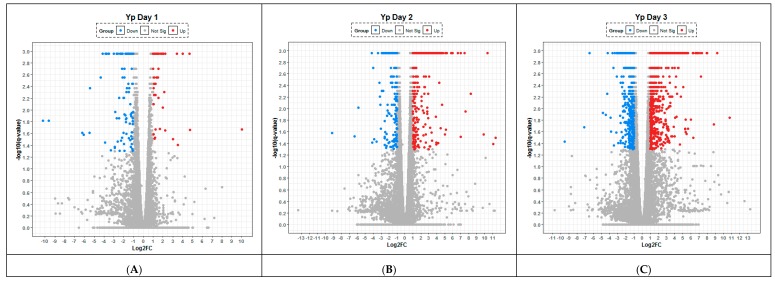
Lung transcriptome following aerosol infection of Balb/C mice with *Yersinia pestis*. Temporal changes are presented after (**A**) 24, (**B**) 48 and (**C**) 72 h following infection with the organism. Any gene that has log-fold change less than 2 versus uninfected control are labelled in grey, significantly up- (red) and down-regulated (blue). The statistical significance (−log10 of the *p*-value) of each gene within the transcriptome is shown on the *y*-axis.

After 24 h p.i. with *Y. pestis*, there was a clear trend towards the downregulation of transcripts (specifically CPA1, CELA1, PNLIPRP1, CPB1) encoding peptidases and elastases involved in the maintenance and/or remodelling of the lung architecture and the associated extracellular matrix. The inert immunological nature of the murine response to *Y. pestis* can clearly be visualised at 24 h p.i. by the small numbers of both up (232 statistically significant) and down regulated (357) transcripts on the volcano plot (Figure 3). However, it is also noticeable at 48 h p.i. these same transcripts also featured in the 10 most upregulated (out of a total of 950) along with a further elastase transcript (CELA3B) (Table 2). There was further upregulation of transcripts for genes influencing antimicrobial activity, either through the production of chemokines involved in cellular recruitment/migration (i.e., CXCL9, CXCL10, IDO1), immune responsiveness (IRG1) and activation of the inflammasome (i.e., GBP5); all were significantly upregulated within the transcriptome on 48 and 72 h p.i. 

**Figure 4:**
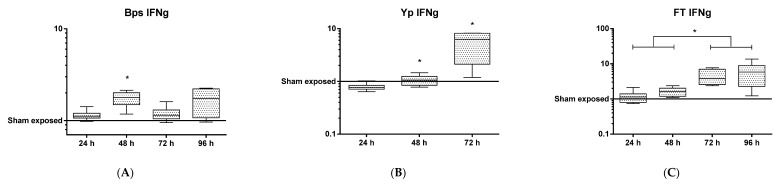
Protein levels of IFNγ in lung tissue following exposure to either (**A**) *Y. pestis*, (**B**) *B. pseudomallei* and (**C**) *F. tularensis* through time. Statistical significance was determined by the Kruskall–Wallis test with Dunn’s multiple comparisons test (* *p* < 0.05).

At the protein level, it is noteworthy that a number of proinflammatory chemokines and cytokines (e.g., CCL2 or MIP1a, GM-CSF) were reduced compared to the control samples (Table S1). Despite significant upregulation of the transcript for the gene encoding IFN-γ early (24 h p.i.) during infection, the expression of this protein in the lung was delayed, appearing in significant quantities only at 72 h (Figure 4), indicating that *Y. pestis* actively inhibited IFN-γ translation. As IFN-γ is known to be important for clearance of both bacterial and viral intracellular pathogens from phagocytic cells, it can be hypothesised that these observations are immunomodulatory mechanisms that aim to enhance the colonisation of host cells during early infection with *Y. pestis*. In contrast, at 48 and 72 h p.i. it was found that the expression of RANTES, IL-12p40 (Table S1) and CXCL1 were upregulated indicating an initiation of the host response to infection (Figure 2). 

### 2.4. F. tularensis Induces a Delayed Activation of the Innate Immune Response 

**Figure 5:**
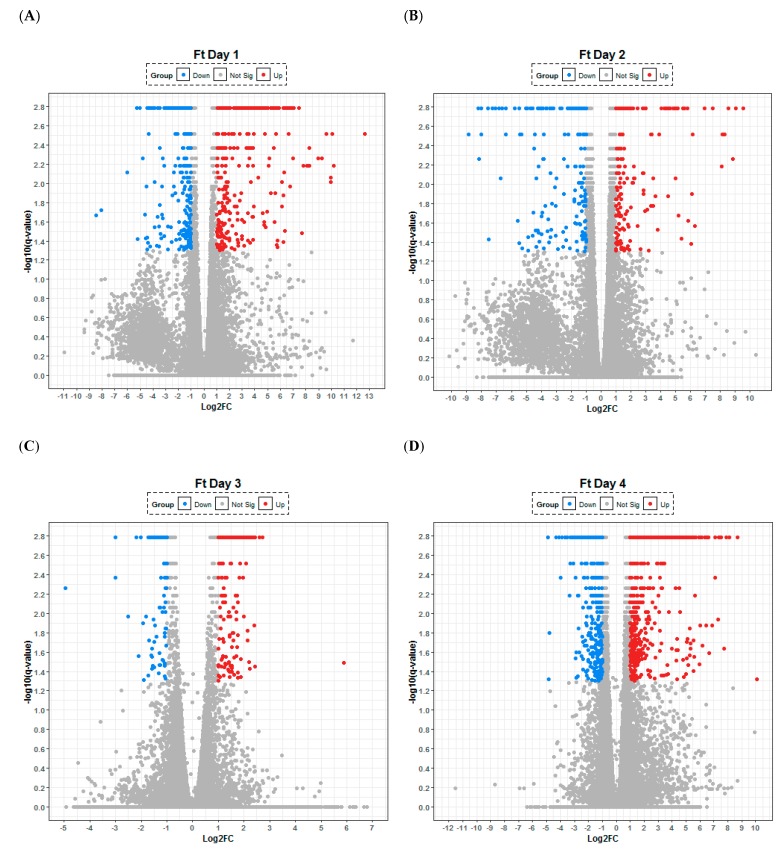
Lung transcriptome following aerosol infection of Balb/C mice with *Francisella tularensis*. Temporal changes are presented after (**A**) 24, (**B**) 48, (**C**) 72 and (**D**) 96 h following infection with the organism. Any gene that has log-fold change less than 2 versus uninfected control are labelled in grey, significantly up- (red) and down-regulated (blue). The statistical significance (−log10 of the *p*-value) of each gene within the transcriptome is shown on the *y*-axis.

Within the first 24 h of infection with *F. tularensis* there was a significant increase in several transcripts although a clear pattern is difficult to decipher (Table 3). Transcripts related to the blood (albumin) and endothelium were up-regulated (i.e., ALB, FILIP1L) along with those involved in metabolism of a variety of compounds (SULT2A1 and CYP3A5). At 24 h, p.i. genes involved in DNA replication and repair (NCPD2, TRRAP and PURA) are down regulated (Table 3). These transcripts continue to be downregulated at 48 h p.i. indicating that *F. tularensis* continued to inhibit these biological processes for up to two days after challenge. *Francisella* is known to be a stealth pathogen avoiding the host immune response during the initial phase of infection. The confused nature of the host response is in part reflected by the volcano plots that highlight that similar numbers of transcripts are up and down regulated at 24 h (369 and 427, respectively) and 48 h p.i (498 and 524, respectively) (Figure 5). 

Consistent with this picture at the transcript level is the delayed expression of arrival of proinflammatory cytokines (e.g., CXCL1, IFNγ) (Figure 2; Table S1). This is supported here with upregulation of immune transcripts, for example, CXCL6, NR4A3 and SAA2-SAA4, becoming apparent at 48 h p.i. Immune related transcripts (ORM1, GM12250, SAA3, BGP5, CXCL9 and CD274) then dominated the majority of the top 10 upregulated transcripts at 72 h p.i., as the classical innate immune response to infection developed. At 48 h p.i. increased protein turnover was indicated by increases in transcripts associated with ubiquitination (RNF149 and UBC). This, coupled with the downregulation of DNA replication and repair transcripts at this time point, may suggest that transcript and protein generation/degradation processes were undergoing significant turnover during the first 48 h of infection, as the host and pathogen compete for dominance and survival.

### 2.5. Common Downregulated Responses during the Acute Phase (24 and 48 h p.i.) of Infection

**Figure 6:**
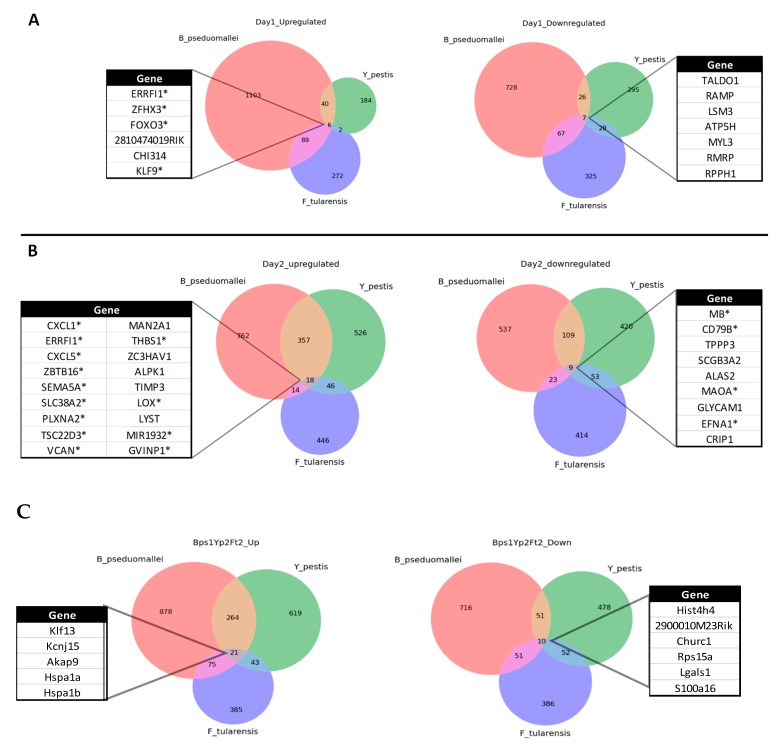
Common host responses in the Balb/C mouse during the acute phase of pulmonary infection with *Burkholderia pseudomallei*, *Francisella tularensis* and *Yersinia pestis* that are upregulated and downregulated on (**A**) 24 h p.i., (**B**) 48 h p.i. or (**C**) additional transcripts identified during a “synchronised” infection (i.e., *B. pseudomallei* at 24 h p.i., *F. tularensis* and *Y. pestis* both at 48 h p.i.). * Indicates common transcripts that are present in both temporal and “synchronised” infections.

We examined whether commonalities existed in the host transcriptomic responses induced within the lungs through time following infection with these three highly virulent bacterial pathogens (Figure 6). Of the downregulated transcripts at 24 h p.i., the associated genes were involved in nucleic acid synthesis (i.e., TALDO1, RMRP), calcium ion binding/absorption (i.e., RAMP, MYL3) and/or protein synthesis (i.e., LSM3, ATPSH, RPPH1) (Figure 6A). The common upregulated transcripts were transcription factors KLF9 (general regulatory element), FOXO3 (linked to apoptosis), ZFHX3 (thought to have a role in inhibiting the cell cycle) and CHI314 (which has been linked to intracellular infection and a Th2 inflammatory response). Collectively, these responses suggest the host is slowing critical cellular functions through downregulation of key components within the lungs as well as a corresponding upregulation of genes that modulate key intracellular functions and/or apoptosis. 

At 48 h p.i., of a total of 66 gene commonalities from the combined transcriptomes, only 9 were found to be consistently downregulated across the three infections considered here (Figure 6B). Of particular interest, is the apparent downregulation of transcripts for genes involved in critical aspects associated with the blood circulatory system, specifically MB (encoding myoglobin), ALAS2 (important for iron binding in haemoglobin) and EFNA1 (involved in erythropoiesis as well as in contact-dependent signalling within neuronal, vascular and epithelial cells). Downregulation of SCGB3A2 (lung surfactant) and TPPP3 (bind tubulin and microtubulin) which are collectively involved in maintaining cell macrostructure and overall tissue integrity is also noteworthy; perhaps aiding the growth, proliferation or dissemination of the bacteria within the lung and the circulatory system, beyond the primary foci of infection.

Finally, at the protein level it was notable that RANTES expression was down regulated and/or absent for all pathogens; despite being significantly upregulated at the transcriptional level (Table S5).

### 2.6. Common Upregulated Responses during the Acute Phase (24 and 48 h p.i.) of Infection

It is noteworthy that only one molecule, ERRFI1 (ErbB receptor feedback inhibitor 1), was consistently upregulated across both the 24 and 48 h p.i. time points. ERRFI1 is thought to inhibit the autophosphorylation of the EGFR family and the downstream signaling proteins, principally in the MAPK, AKT and JNK pathways, ultimately inhibiting DNA synthesis and cell proliferation (i.e., a potential further contributor to the slowing down of critical cellular functions). At 24 h p.i the small numbers of common transcripts are largely associated with transcriptional regulators/factors (e.g., FOXO3, Klf9, Zfhx3). However, of the 18 upregulated genes common to the three infections at 48 h p.i., a third are involved in maintaining tissue integrity and/or cellular macrostructure (Figure 6). The SEMA5A (tissue remodelling, F-actin assembly), PLXNA2 (invasive growth and cell migration) and LOX (role in fibrous collagen and elastin development) are involved in the physical structure of tissues, whilst THBS1 (glycoprotein in cell–cell and cell–matrix interactions), VCAN (cellular macrostructure and major component of the extracellular matrix) and TIMP3 (inhibition of matrix metalloproteases and extracellular matrix) are important for the integrity of the mucosal surface of the lung. Intracellular transport/trafficking/degradation is a second theme of the upregulated genes. Specifically, LYST regulates intracellular protein trafficking in endosomes, ZBTB16 is involved in marking proteins for degradation through the E3 ubiquitin protein ligase complex and MANZA1 is involved in Golgi formation and sugar conversion. It is conceivable that these changes in gene expression represent, considering the rapidity of disease progression, a late response of the host which is heavily infected with intracellular bacteria. Supporting this theory, is the prevalence of upregulated GVIN1 (interferon-induced GTPase) and ZC3HAV1 (associated with mRNA turnover), both of which have previously been linked to an intracellular response to invading pathogens including viruses but here may constitute a response to an intracellular bacterial infection [25,26].

Commonalities in protein expression across the pathogens was predominantly associated with IL-6 expression; whilst limited observable differences noted for other Interleukins, specifically IL-2, -3, -4 or -5 (Table S1).

### 2.7. Early Host Responses Indicate the Lung Is Immunologically and Metabolically Dormant during Infection with F. tularensis and Y. pestis, but not B. pseudomallei

Following *B. pseudomallei* infection, the top 5 most upregulated pathways between 24 and 72 h p.i. remain fairly consistent (Table S2). At 24 h p.i. it is clear that the host has recognised an insult by triggering the acute phase response pathway which provides a non-specific defence mechanism against microorganisms. In addition, the IL10 signalling pathway is in the top 4 most upregulated canonical pathways on every day of the *B. pseudomallei* infection time course. At 48 h p.i. the pathway involving pattern recognition receptors for bacteria and viruses appears in the top 10 upregulated pathways and remains there for the rest of the time course providing some evidence that the host is trying to control the infection.

In contrast to early *B. pseudomallei* infection, the lung appeared to be in a relatively immunologically and metabolically dormant state during the first 24–48 h p.i. with *F. tularensis* and *Y. pestis.* Within the top 5 downregulated pathways it is notable that those involved in innate immune responses and metabolism feature (i.e., oxidative phosphorylation, mitochondrial dysfunction and EIF2 signalling) (Tables S3 and S4). The top 10 upregulated pathways (e.g., epithelial adherens junction, actin cytoskeleton signalling) are associated with the macro-architecture or modulation of the epithelial integrity of the organ. The top three most significantly upregulated canonical pathways; hepatic fibrosis/stellate cell activation, agranulocyte adhesion/diapedesis and granulocyte adhesion/diapedesis highlight the potential for increased cellular influx. However by 72 h p.i. there is notable dysregulation of the host response, comparable to the latter stages of infection with *B. pseudomallei* (Table S2). Most strikingly, during infection with *F. tularensis* the agranulocyte adhesion/diapedesis pathway shifts from being one of the most downregulated pathways at 48 h p.i. to one of the key upregulated pathways from 72 h p.i. onwards (i.e., a day later than observed for *Y. pestis*).

As observed here for *Y. pestis*, the transcriptomic induction of pathways associated with the production of inflammatory chemokines and cytokines in response to *F. tularensis* was delayed. Only after 72 h p.i. do IL-6 and IL-10 pathways feature in the top 10 most upregulated and the appearance of TREM1 signalling pathways highlights the host response has identified the presence of microbial products.

### 2.8. Synchronicity of Infection across the Agents

By examining the commonalities between pathways during each infection time course, it was clear that the data for 24 h p.i. with *B. pseudomallei* mirrored closely that generated at 48h p.i. for *F. tularensis* and *Y. pestis.* Therefore we studied the responses across these “synchronised” infections and identified a number of unique commonalities (Figure 6C). Out of a total of 21 shared upregulated transcripts, five were uniquely identified through this analysis (Klf13, Akap9, hspa1a, hspa1b, kcnj15) and found to be associated with shared pathways. There were 10 common downregulated transcripts with a further six being unique to the synchronised infection (i.e., Hist4h4, 2900010M23Rik, Churc1, Rps15a, Lgals1, S100a16). Of those annotated, most were associated with cell proliferation/division. In particular, Lgals1 is associated with cell–cell and cell–matrix interactions associated with maintain cell/lung integrity as well as intracellular activations processes including apoptosis and cell–matrix interactions.

## 3. Discussion

Our research has fully characterised lung responses following pulmonary infection of Balb/C mice with highly virulent Gram-negative bacteria. We have used statistically-powered in vivo models along with an unbiased RNAseq approach and a bespoke bioinformatics pipeline analysis. For the first time we have identified common genes/pathways in the global gene expression profiles during the acute phase of three different infections, specifically pneumonic plague, melioidosis and tularemia. As well as increasing fundamental knowledge of the biological responses induced by highly pathogenic bacteria, the output has identified points of intervention critical to host survival and will enable the rational design of therapies based on early changes in gene expression.

The data arising from this RNAseq approach complement those gained from microarray/chip-based analyses of the transcriptome and the pathways triggered in response to infection with species from the Genera *Burkholderia*, *Francisella* and *Yersinia* [12,17,22,26,27,28,29,30]. In particular, our data closely mirrors that previously published for a different clinical strain of *B. pseudomallei* within experimental murine (C57BL/6) models of infection and human blood responses [12]. The rapidity of the replication of *B. pseudomallei* from the primary site of infection is well known [9,10] and was observed here (Figure S1). We have also demonstrated that the proliferation of *F. tularensis* and *Y. pestis* within the lung although slower increased exponentially in all cases from 48 h p.i (Figure S1). Within the transcriptome the later time points of 72 and 96 h p.i. at both the gene and pathway level present a confused picture (e.g., upregulation of both pro- and anti-inflammatory cascades/molecules) and therefore offer little utility in understanding the progression of disease as the host attempts to temper rampant infections.

At the earlier time points, it is clear from comparing the lung transcriptomic responses within the mouse that despite the rapid proliferation of bacteria within this primary focus of infection, there is limited immunological recognition by the host. In particular, following challenge with *F. tularensis* and *Y. pestis* a limited and/or ineffective response appears to be mounted towards the developing acute infection. However, by taking a global view through studying the canonical pathways, clear patterns emerge. There is firstly, an early downregulation of critical cellular functions (e.g., mitochondrial dysfunction, oxidative phosphorylation) with a subsequent upregulation of pathways involved in maintaining tissue integrity/function (e.g., granulocyte adhesion and diapedesis). There is also evidence of activation of an ultimately futile, immune response via the upregulation of both IL-6 (a pleiotropic cytokine) and IL-10 (a predominantly anti-inflammatory) signalling pathways (Tables S2–S4); perhaps signifying the host’s attempt to maintain immune homeostasis in the face of pathogen-induced dysregulation [31]. Consistent with previously published research [9,15,32], the secretion of IL-10 (a key inflammatory mediator IL-10 [33]) was absent during the early stages of infection with all 3 pathogens (Table S1). This suggests that the host is trying to maintain an immunological balance within the transcriptome that does not translate to the proteome, leading to a polarised pro-inflammatory response that can cause significant host damage and is detrimental to survival as observed during other infectious diseases (e.g., Influenza A) [34].

At an individual gene level, the commonalities in the host response fall into four distinct categories across the acute phase of infection up to 48 h post-challenge. The downregulation of (1) critical cellular functions (e.g., DNA synthesis, cell proliferation, apoptosis), (2) components of the circulatory system (e.g., myoglobin, heme-binding protein production); or alternatively, the upregulation of genes encoding transcripts involved in (3) maintaining tissue integrity (e.g., cellular cytoskeletons, major components of the extracellular matrix) or (4) intracellular regulatory processes (e.g., golgi formation/trafficking, mRNA turnover). As each pathogen has an intracellular phase in its life cycle, one might expect to see regulatory processes affected and/or modulated during infection. Equally, tissue integrity is clearly a key component in preventing bacterial pathogen spread and dissemination. We hypothesise from the downregulation of blood circulatory component transcripts (e.g., MB, ALAS2 and EFNA1) demonstrated here that such attempts at protective responses have been unsuccessful.

Further, even though infection was commonly found to induce a variety of transcripts for chemokines involved in cellular recruitment it is notable either here (Table S1) or through previous studies [9,12,15,32] that there is a demonstrable reduction in RANTES expression early in the time course of infection in the mouse consistent with previous research [35] (Figure 2). This is significant as the downregulation of RANTES has previously been suggested as an early indicator of sepsis in humans [36] suggesting a more active modulation of cellular recruitment to the lung by the bacteria at the protein rather than the transcript level. Adding further evidence in support of this theory is our unique analysis of comparing “synchronised” infections (24 h for *B. pseudomallei* and 48 h for *F. tularensis* and *Y. pestis*) which revealed a number of additional common molecules, in particular, the upregulation across all agents of the transcription factor Kruppel like factor 13, KLf13 (Figure 6). We also noted the sustained upregulation of the transcript Errfi1 (MIG6 or RALT) across both 24 and 48 h post-infection for all three pathogens (Figure 6); although not in the top 10 upregulated transcripts (Table 1, Table 2 and Table 3) where it was the only molecule common during all three infections. This molecule is a negative regulator of the mitogenic (MAPK) cascade, inhibiting tyrosine kinases within the Epidermal Growth Factor Receptor (EGFR) pathway associated with cell proliferation and the prevention of carcinogenesis [37]. Errfi1 is thought to help maintain cell/tissue homeostasis through growth inhibition, cell senescence and apoptosis [38]. Although the role of Errfi1 in tumour repression is well documented, to the authors’ knowledge there is no previous evidence that highlights a key role for this molecule during infection and therefore this may represent a new line of investigation in the treatment or diagnosis of acute infections

Finally, this research has also revealed interesting aspects of the progression of disease for each individual pathogen. We demonstrated that each pathogen alters the host response at either the transcriptomic or proteomic level up to 48 h p.i. It is well documented that *F. tularensis* suppresses immune activation to allow survival in its intracellular niche [21,28]. For *Y. pestis*, two main stories emerge from the analysis of the transcriptome at the individual gene level following infection. Firstly, a number of the significantly downregulated transcripts after 24 h are also the most upregulated after 48 h (Table 2). Secondly, the pathway analysis of the transcriptomics data revealed that after 24 h, IFN-γ was the most significantly upregulated transcript, but that this did not translate into protein expression (Figure 4). Modest increases in IFN-γ during infection with *Y. pestis* have previously been reported along with the late arrival of TNFα, also demonstrated here [15,39]. Both TNFα and IFNγ are thought to be critical for T cell-mediated protection against *Y. pestis* in pulmonary models of *Y. pestis* [40]. Activated macrophages produce TNFα however as this molecule is only present in significant quantities at 72 h p.i., when half of the animals within this group have succumbed to the disease, it would appear this is “too little, too late”. In addition, it is noteworthy that various other proinflammatory cytokines involved in antimicrobial responses (i.e., IL-2, IL-3, IL-10) are absent or are produced at minimal levels across all three days of the study (Table S1). Like the previously published data for *F. tularensis*, we provide further evidence here of modulation at the translational level of key proinflammatory cytokines, contributing to the virulence of *Y. pestis* as a stealth pathogen. This also extends to proteins involved in cellular recruitment, specifically CXCL1/KC, a chemokine involved in neutrophil attraction, which is expressed early and at high levels during a rampant *B. pseudomallei* infection (Figure 2). However, this molecule is largely absent during *Y. pestis* and *F. tularensis* infections and perhaps aids the survival and dissemination of these slower growing organisms (Figure 2).

## 4. Conclusions

This research has characterised the early responses within the lung during the acute pulmonary infections that are caused by three specific highly virulent Gram-negative bacteria. For the first time, a series of common pathways that are associated with the (a) slowing down of critical cellular functions, (b) production of circulatory system components, (c) lung tissue integrity and (d) intracellular regulatory processes have been identified that are key to the development of the diseases caused by each organism. Additionally, a single gene Errfi1 was found to be upregulated in the transcriptome during all infections; potentially representing a unique marker of acute infection. Whilst post-transcriptional modulation of RANTES expression was also found to occur across all pathogens with one hypothesis being these infections directly or indirectly modulate cell trafficking through chemokine expression/detection. Finally, through this research, pathogen-specific temporal changes in the host response have been identified which may provide new mechanistic data on the progression of the diseases caused by *B. pseudomallei*, *F. tularensis* and *Y. pestis*. Ultimately, these studies open up the potential for completely new therapeutic interventions that can either mitigate pathogen-induced modulation of the host response or, alternatively, that can bring a dysregulated immune response back to homeostasis, increasing the prospect of survival following acquisition of these highly acute infectious diseases.

## 5. Materials and Methods

### 5.1. Bacteria

*B. pseudomallei* strain K96243 was originally recovered from a case of human melioidosis in Thailand [41], whilst *F. tularensis* SchuS4 and *Y. pestis* CO92 were originally from human cases in the USA of tularemia and pneumonic plague, respectively [42,43].

### 5.2. Aerosol Challenge of Animals

All animal studies were carried out in accordance with the Animal (Scientific Procedures) Act (1986). In order to monitor the progress of infection with time, groups (n = 10) of animals were culled at 24 h, 48 h, 72 h and 96 h post-infection and bacterial loads were enumerated in the lung. Challenge doses were chosen for each infectious disease with the aim of ensuring survival of animals out to 96 h however due to the severity of a pulmonary infection with *Yersinia pestis* this study ended at the 72 h time point. Consistent across all studies and time points a group of control animals (n = 10), housed as per the infected animals but receiving a PBS aerosol spray and were also culled to provide baseline data. Humane end points were strictly observed which led to the culling of some mice prior to their assigned time point.

Female, 8–10 weeks old Balb/C mice (Charles River Laboratories) were acclimatised to the facility for 7 days, prior to being exposed to pathogen by the aerosol route using a Collison nebulizer and modified Henderson apparatus as described by Lever et al. (2009) [10]. *B. pseudomallei* was prepared for the animal challenge by growing for 20 h in Luria Broth at 37 °C on a rotary platform. The overnight culture was then diluted in PBS to an OD_590_ of 0.39 and a 1 in 100 dilution of this was used in the Henderson apparatus to generate the challenge aerosol (calculated as 5.5 × 10^5^ CFU/mL). Mice were exposed for 10 min, and impinger samples were collected for 1 min during each exposure enabling viability counting. During challenge studies with *B. pseudomallei* K96243, surviving animals at each time point are indicated in parenthesis 24 h (10), 48 h (8), 72 h (9) and 96 h (4). The dose retained by each mouse was calculated [44,45] to be an average of 30 CFU. *Y. pestis* CO92 was prepared for the animal challenge by growing for 48 h in blood agar base (BAB) broth at 28 °C on a rotary platform. The retained dose of *Y. pestis* for each mouse was calculated [44,45] and an average of 1.2 × 10^4^ CFU/mL determined; remaining animal numbers are shown in parenthesis at 24 h (10), 48 h (10) and 72 h (6). *F. tularensis* was grown and prepared for challenge as described previously by Hamblin et al. (2014) [46]. The challenge dose of *F. tularensis* was calculated as 5 × 10^8^ CFU/mL and the dose retained by each mouse [44,46] was an average of 45 CFU; surviving animals are indicated in parenthesis 24 h (9), 48 h (10), 72 h (10) and 96 h (9). Across all three challenge studies at the set time points of 24 h, 48 h, 72 h and 96 h p.i. groups of 10 mice were terminally anesthetised and exsanguinated by intracardial puncture. The lung was removed, weighed and cut in half. Half was placed in RNA*later* (Qiagen) and stored at −20 °C prior to RNA extraction and the other half was homogenised in PBS by passing through a 40 µm cell strainer (BD Falcon) and used for enumeration of the bacteria. The number of viable bacteria per organ was determined by serially diluting each lung tissue homogenate in PBS followed by cultivation of 0.1 mL in triplicate for a minimum of 24 h.

### 5.3. RNA Extraction and Library Preparation

RNA was extracted from lung tissue and analysed by RNASeq before being put through the developed bioinformatics pipeline. The lung samples stored in RNA*later* werehomogenised in Qiagen buffer RLT using a Bullet Blender^®^ (Next Advance, Inc.) with 100 µL RNase-free SSB14B beads on speed 8 for 4 min, followed by speed 10 for 3 min. RNA was extracted from the equivalent of 10 mg of tissue using the RNeasy mini kit animal tissues protocol (Qiagen), followed by DNA removal using Invitrogen TURBO DNA-*free*™ (Thermo Fisher Scientific). RNA integrity and concentration were assessed using an Agilent 2100 Bioanalyzer (Agilent Technologies). ERCC Exfold RNA Spike-In Control mix (Ambion^TM^ at Thermo Fisher Scientific; Spike-In Mix 1 and 2 were randomised between the samples) was added to each sample before using a Ribo-Zero rRNA removal kit Human/Mouse/Rat (Illumina). Libraries were prepared for sequencing using ScriptSeq™ v2 RNA-seq library preparation kit (Illumina) and subjected to paired-end RNA-seq on an Illumina HiSeq2500.

### 5.4. Data Analysis Pipeline

Reads were cleaned using the fastq-mcf pipeline from the eautils toolkit (https://expressionanalysis.github.io/ea-utils/). Ribosomal reads were removed by mapping to a set of representative ribosomal sequences using Bowtie 2.2. Remaining non-ribosomal reads were mapped to the mouse genome (mm10 reference genome) using TopHat v2.0.4 [47], and gene expression values were calculated using Cufflinks v2.0.2 [48]. Differentially expressed genes between infected and control mice were identified by statistical analysis (CuffDiff). These data were then probed for biological significance using Ingenuity Pathway Analysis software (Qiagen Ltd.). For pathway analysis, the Fisher’s exact test was used to assess significance (*p* < 0.05) of the association of the genes in the data set with canonical pathways within the Ingenuity Knowledge Base (Qiagen Ltd.) The actual fold changes of all up- or down regulated transcripts for each bacterial infection has also been made available (Table S5). Volcano plots were produced in R (version 3.4.3).

### 5.5. Statistical analysis

Statistical analysis of RNASeq data was performed as outlined in the Data analysis pipeline section of these materials and methods. All other data was presented graphically using Graphpad PRISM v6. Statistical analysis of RANTES, Cxcl1 and IFNγ was determined by the Kruskall–Wallis test with Dunn’s multiple comparisons test. Bacterial load data were transformed by the logarithm of 10 in order to fit the normal distribution and statistical significance determined by ANOVA with Tukey’s multiple comparison. Lung wet weights were analysed by 2-way ANOVA with Sidak’s multiple comparison test.

### 5.6. Quantification of inflammatory molecules (Luminex)

Following the enumeration of bacteria, in order to measure the quantities of cytokines and chemokines present within tissues, 200 uL aliquots of homogenate were centrifuged for 5 min at 2000 rpm. Supernatants were removed for cytokine analysis and stored at - 80 °C prior to analysis. Luminex (Bio-plex^®^; 23-plex mouse inflammatory cytokine kit) assay was carried out in accordance with manufacturer’s instructions (Bio-Rad™) and using the Bio-plex^®^ 200 reader. Fluorescent readings from all individual biological replicates per time-point for each agent were normalised to the arithmetic mean fluorescent value of three time and agent matched control samples. Data in the table is expressed as the arithmetic mean of this control normalised value.

## Figures and Tables

**Table 1:** The top 10 statistically significant up regulated and down regulated transcripts on each day of the infection involved in the response to *B. pseudomallei* infection in the mouse lung when compared to uninfected mice. Log ratio is expressed as the Log2 expression values of infected versus control samples.

**Up Regulated Genes in *B. pseudomallei* Infection**							
**24 h** **Gene**	**24 h** **Log Ratio**	**48 h** **Gene**	**48 h** **Log Ratio**	**72 h** **Gene**	**72 h** **Log Ratio**	**96 h** **Gene**	**96 h** **Log Ratio**
IRG1	8.032	IRG1	10.311	IRG1	11.341	CXCL3	11.258
CXCCL11	7.133	SAA3	9.862	NGP	9.948	STFA2	9.675
PNLIP	7.129	CXCL6	8.853	STFA2	9.906	IL6	9.489
CXCL10	6.796	IL6	8.560	CXCL3	8.874	STFA1	9.179
PTX3	6.504	SAA1	8.466	SAA3	8.738	CCL3L3	8.977
CXCL9	6.370	IDO1	8.057	CSTA	8.187	CXCL6	8.939
CCL7	6.361	CXCL3	7.737	IL6	8.055	CXCL2	8.928
CPA1	6.213	IL17A	7.350	LY6A	7.865	SAA3	8.396
CXCL6	5.874	CXCL9	7.029	CXCL6	7.763	IFITM6	8.381
CXCL2	5.833	CXCL2	6.964	CCL3L3	7.673	IL1R2	8.330
**Down Regulated Molecules in *B. pseudomallei* Infection**							
**24 h** **Gene**	**24 h** **Log ratio**	**48 h** **Gene**	**48 h** **Log ratio**	**72 h** **Gene**	**72 h** **Log ratio**	**96 h** **Gene**	**96 h** **Log ratio**
MIR-467	−4.237	MIR-467	−4.520	GHRL	−6.653	MT4	−4,773
FAIM3	−2.489	ESM1	−3.957	ALB	−5.950	COMMD1	−4.611
MYL2	−2.187	TTR	−3.935	MYL2	−4.651	MYL3	−4.414
ZNF488	−2.187	ALB	−3.126	CYP1A1	−4.383	CRABP2	−4.332
TNNC2	−1.870	DBP	−3.003	THRSP	−4.029	CCL25	−4.322
ANGPTL7	−1.837	ALDH3A1	−2.879	GRP	−4.013	CALML5	−4.317
PCK1	−1.825	LCE1C	−2.819	KRT10	−3.731	RPTN	−4.162
CTCFL	−1.794	ASGR1	−2.785	COLQ	−3.464	UDGT2B10	−4.045
RPL13A	−1.757	CYP2A6	−2.763	PLA2G1B	−3.415	TGM3	−4.034
ROGD1	−1.734	APLNR	−2.677	SLC7A10	−3.255	TTR	−3.943

**Table 2:** The top 10 statistically significant up regulated and down regulated genes on each day of the infection involved in the response to *Y. pestis* infection in the mouse lung when compared to uninfected mice. Log ratio is expressed as the Log2 expression values of infected versus control samples.

**Up Regulated Genes in *Y. pestis* Infection**					
**24 h** **Gene**	**24 h** **Log Ratio**	**48 h** **Gene**	**48h** **Log Ratio**	**72 h** **Gene**	**72 h** **Log Ratio**
IFNγ	3.611	CELA3B	9.862	CXCL9	9.528
CD3EAP	2.217	CXCL10	8.293	CXCL3	8.807
CYP26B1	1.959	CPA1	7.605	CXCL2	8.028
HIST1H2A	1.794	CXCL9	7.484	MT2	7.920
ZBTB16	1.765	IRG1	7.008	CCL2	7.341
VMN1R63	1.693	IDO1	6.625	C15orf48	7.213
Gm4851	1.556	CELA1	6.003	SAA3	7.088
TCF7L2	1.500	GBP5	5.810	MT1	6.874
CD177	1.479	PNLIPRP1	5.282	MMP8	6.769
SYT11	1.442	CPB1	5.166	GBP5	6.600
**Down Regulated Genes in *Y. pestis* Infection**					
**24 h** **Gene**	**24 h** **Log Ratio**	**48 h** **Gene**	**48 h** **Log Ratio**	**72 h** **Gene**	**72 h** **Log Ratio**
CTRB2	−10.237	GSDMA2	−9.141	LRRC17	−3.632
PRSS3	−9.629	PSCA	−5.864	SFRP2	−3.196
CPA1	−6.194	MCTP1	−4.110	COLQ	−3.169
CELA1	−5.422	UGT2B10	−3.974	COMMD1	−3.080
KRT1	−4.352	SULT1B1	−3.875	APLNR	−3.026
PNLIPRP1	−4.159	DNTT	−3.375	CPA3	−2.898
CPB1	−3.740	KRT1	−2.890	MYCT1	−2.877
RNASE1	−3.013	RASA2	−2.560	CYP1A1	−2.674
RNU12	−3.012	CLPS	−2.520	C1QTNF2	−2.674
STFA1	−2.276	COMMD1	−2.487	DBP	−2.641

**Table 3:** The top 10 statistically significant up regulated and down regulated genes on each day of the infection involved in the response to *F. tularensis* infection in the mouse lung when compared to uninfected mice. Log ratio is expressed as the Log2 expression values of infected versus control samples.

**Up Regulated Genes in *F. tularensis* Infection**							
**24 h** **Gene**	**24 h** **Log Ratio**	**48 h** **Gene**	**48 h** **Log Ratio**	**72 h** **Molecule**	**72 h** **Log Ratio**	**96 h** **Gene**	**96 h** **Log Ratio**
SULT2A1	10.051	FILIP1L	7.471	KRT1	5.880	PTPRV	10.085
G6PC	9.581	DYNLT1	5.191	ORM1	2.725	IDO1	8.697
MUP1	8.282	LARS2	4.225	GM12250	2.599	SAA3	8.688
FILIP1L	7.062	CXCL6	2.785	SAA3	2.330	CCL7	7.496
TERC	7.010	RNF149	2.469	GBP5	2.264	NGP	7.364
BHMT	6.970	NR4A3	2.211	SYCP2	2.263	CXCL10	7.314
TTR	6.867	SAA2SAA4	2.130	CXCL9	2.249	CXCL9	7.095
CYP3A5	6.859	UBC	1.828	IDO1	2.220	IL6	7.070
DYNLT1	6.785	ERBB4	1.738	WARS	2.219	ORM1	6.663
ALB	6.677	CHRM2	1.689	CD274	2.213	GM12250	6.617
**Down regulated molecules in *F. tularensis* infection**							
**24 h** **Gene**	**24 h** **Log Ratio**	**48 h** **Gene**	**48 h** **Log Ratio**	**72 h** **Gene**	**72 h** **Log Ratio**	**96 h** **Gene**	**96 h** **Log Ratio**
NCAPD2	−8.452	NCAPD2	−8.233	MYL2	−4.953	APLNR	−4.904
TRRAP	−8.080	TRRAP	−8.169	PVALB	−3.018	ESM1	−4.336
PURA	−4.420	GUCA2B	−5.476	CLCA1	−2.508	KRT1	−3.866
LRRC8B	−4.195	MYL2	−5.475	RPS16	−2.178	SCN3A	−3.653
PTMS	−3.896	NSUN3	−4.869	CCR3	−2.007	CCR3	−3.632
CEL	−3.643	RNF141	−4.383	NPPB	−1.932	GLP1R	−3.339
2210010C04	−3.621	PTCH1	−4.352	UCP1	−1.805	ZMAT4	−3.299
PNLIP	−3.571	PURA	−4.229	ALB	−1.740	MFAP5	−3.282
NEBL	−3.542	KIAA1147	−4.111	ESM1	−1.716	FCRLS	−3.191
CLPS	−3.504	LRRC8B	−4.002	NPPA	−1.625	CYP1A1	−3.134

## Supplementary Materials

The following are available online at https://www.mdpi.com/2076-0817/8/4/159/s1, Figure S1: Bacterial load per gram of mouse lung tissue following infection with *B. pseudomallei*, *F. tularensis* and *Y. pestis*. Statistical significance was determined by ANOVA with Tukey’s multiple comparison (* *p* < 0.05), Figure S2: Clinical signs indicate progression of disease severity with time for *B. pseudomallei, F. tularensis and Y. pestis*. Mice were monitored over time and scored on severity from 0 (no clinical signs), 1 (mild piloerection + normal mobility), 2 (medium piloerection + normal mobility), 3 (severe piloerection + normal mobility), 4 (severe piloerection + limited/reduced mobility) to 5 (severe piloerection + unable to move). B: Lung wet weight following infection with *B. pseudomallei*, *F. tularensis* and *Y. pestis*. Statistical significance was determined by 2-way ANOVA with Sidak’s multiple comparison test (* *p* < 0.05). Table S1: Inflammatory cytokine data (supplied as a separate file). Table S2. The most statistically significant up and down regulated pathways across the time course of infection in the mouse lung infected with *B. pseudomallei* by the aerosol route. Table S3. The most statistically significant up and down regulated pathways across the time course of infection in the mouse lung infected with *F. tularensis* by the aerosol route. Table S4. The most statistically significant up and down regulated pathways across the time course of infection in the mouse lung infected with *Y. pestis* by the aerosol route. Table S5. Complete list of statistically significant up- or downregulated transcripts within all three bacterial infections (supplied as a separate file).

## Notes

**Nucleotide sequence accession number.** The RNA sequencing reads are in the process of being deposited.

## Notes

Content includes material subject to © Crown copyright (2019), Dstl. This material is licensed under the terms of the Open Government Licence except where otherwise stated. To view this licence, visit: http://www.nationalarchives.gov.uk/doc/open-government-licence/version/3 or write to the Information Policy Team, The National Archives, Kew, London TW9 4DU, or email: psi@nationalarchives.gsi.gov.uk.
