# Supplementary material for: Common Host Responses in Murine Aerosol Models of Infection Caused by Highly Virulent Gram-Negative Bacteria from the Genera Burkholderia, Francisella and Yersinia
Source: Pathogens. 2019 Sep 21;8(4):159. doi: 10.3390/pathogens8040159 (PMC6963870; doi:10.3390/pathogens8040159)
Supplement: Supplementary file 1 [file pathogens-08-00159-s001.zip › pathogens-543308 supplementary/pathogens-543308 supplementary_doc1.docx]

Supplementary Data 1

**Figure S1:** Bacterial load per gram of mouse lung tissue following infection with *B. pseudomallei*, *F. tularensis* and *Y. pestis*. Statistical significance was determined by ANOVA with Tukey’s multiple comparison (* p < 0.05).

**Figure S2:** Clinical signs indicate progression of disease severity with time for *B. pseudomallei*, *F. tularensis* and *Y. pestis*. Mice were monitored over time and scored on severity from 0 (no clinical signs), 1 (mild piloerection + normal mobility), 2 (medium piloerection + normal mobility), 3 (severe piloerection + normal mobility), 4 (severe piloerection + limited/reduced mobility) to 5 (severe piloerection + unable to move). **B:** Lung wet weight following infection with *B. pseudomallei*, *F. tularensis* and *Y. pestis*. Statistical significance was determined by 2-way ANOVA with Sidak’s multiple comparison test (* p < 0.05).
