# Supplementary material for: Common Host Responses in Murine Aerosol Models of Infection Caused by Highly Virulent Gram-Negative Bacteria from the Genera Burkholderia, Francisella and Yersinia
Source: Pathogens. 2019 Sep 21;8(4):159. doi: 10.3390/pathogens8040159 (PMC6963870; doi:10.3390/pathogens8040159)
Supplement: Supplementary file 1 [file pathogens-08-00159-s001.zip › pathogens-543308 supplementary/pathogens-543308 supplementary_doc2 .docx]

Table S1. Inflammatory cytokine data (supplied as a separate file)

**Table S2.** The most statistically significant up and down regulated pathways across the time course of infection in the mouse lung infected with *B. pseudomallei* by the aerosol route.

| **.** | **Top pathways associated with up-regulated genes in *B. pseudomallei* infection** | | | |
| --- | --- | --- | --- | --- |
|  | **24 h p.i.** | **48 h p.i.** | **72 h p.i.** | **96 h p.i.** |
| 1 | Granulocyte Adhesion and diapedesis | Granulocyte Adhesion and diapedesis | Granulocyte Adhesion and diapedesis | IL-10 Signalling |
| 2 | IL-10 Signalling | Role of Pattern recognition receptors in recognition of bacteria and viruses | Agranulocyte Adhesion and diapedesis | Granulocyte Adhesion and diapedesis |
| 3 | Acute phase response signalling | Type 1 diabetes melitus signalling | IL-10 Signalling | IL-6 signalling |
| 4 | Glucocorticoid receptor signalling | IL-10 Signalling | Role of Pattern recognition receptors in recognition of bacteria and viruses | Acute phase response signalling |
| 5 | Agranulocyte Adhesion and diapedesis | Dendritic Cell maturation | TREM1 signalling | Production of nitric oxide and reactive oxygen species in macrophages |
| 6 | Nfk-b Signalling | Agranulocyte Adhesion and diapedesis | Production of nitric oxide and reactive oxygen species in macrophages | Fcγ receptor-mediated phagocytosis in macrphages and monocytes |
| 7 | IL-6 signalling | TREM1 signalling | IL-6 signalling | iNOS Signalling |
| 8 | Role of macrophages, fibroblasts and endothelial cells in rheumatoid arthritis | Altered T cell and B cell signalling in rheumatoid arthritis | Dendritic Cell maturation | B cell receptor signalling |
| 9 | Dendritic Cell maturation | Role of PKR in interferon production and antiviral response | Fcγ receptor-mediated phagocytosis in macrophages and monocytes | Toll-like receptor signalling |
| 10 | TREM1 signalling | Death receptor signalling | Role of macrophages, fibroblasts and endothelial cells in rheumatoid arthritis | Role of Pattern recognition receptors in recognition of bacteria and viruses |
|  | **Top pathways associated with down-regulated genes in *B. pseudomallei* infection** | | | |
|  | **24 h p.i.** | **48 h p.i.** | **72 h p.i.** | **96 h p.i.** |
| 1 | Mitochondrial dysfunction | hepatic fibrosis / hepatic stellate cell activation | Oxidative phosphorylation | Calcium signalling |
| 2 | Oxidative phosphorylation | Axonal guidance signalling | Mitochondrial dysfunction | NRF2-mediated oxidative stress response |
| 3 | Glycolysis 1 | Nicotine degradation 2 | Axonal guidance signalling | Glutathione-mediated detoxification |
| 4 | Leukocyte extravasation signalling | TR/RXR Activation | Glutathione-mediated detoxification | Oxidative phosphorylation |
| 5 | Putrescine degradation3 | LPS/IL-1 mediated inhibtion of RXR function | Protine kinase A signalling | Cellular effects of sildenafil (Viagra) |
| 6 | Tight junction signalling | Nicotine degradation 3 | Factors promoting cardiogenesis in vertebrates | Mitochondrial dysfunction |
| 7 | Sertoli cell-sertoli cell junction signalling | LXR/RXR Activation | Calcium signalling | Methylmalonyl pathway |
| 8 | hepatice fibrosis / hepatic stellate cell activation | FXR/RXR Activation | hepatice fibrosis / hepatic stellate cell activation | iCOS-iCOSL signalling in T-helper cells |
| 9 | IL-4 signalling | Adipogenesis pathway | Phospholiapse C signalling | Protine kinase A signalling |
| 10 | Oxidative ethanol degradation | Epithelial adherens junction signalling | EIF2 Signalling | Leucine degradation 1 |

**Table S3.** The most statistically significant up and down regulated pathways across the time course of infection in the mouse lung infected with *F. tularensis* by the aerosol route.

|  | **Top pathways associated with up-regulated genes in *F. tularensis* infection** | | | |
| --- | --- | --- | --- | --- |
|  | **24 h p.i.** | **48 h p.i.** | **72 h p.i.** | **96 h p.i.** |
| 1 | FXR/RXR Activation | Axonal Guidance Signaling | Granulocyte Adhesion and Diapedesis | Granulocyte Adhesion and Diapedesis |
| 2 | LXR/RXR Activation | PTEN Signaling | IL-10 Signaling | Acute Phase Response Signaling |
| 3 | Acute phase response signalling | Epithelial Adherens Junction Signaling | TREM1 Signaling | Death Receptor Signaling |
| 4 | Coagulation system | Caveolar-mediated Endocytosis Signaling | Interferon Signaling | Dendritic Cell Maturation |
| 5 | Intrinsic prothrombin activation pathway | Protein Kinase A Signaling | Agranulocyte Adhesion and Diapedesis | IL-6 Signaling |
| 6 | Clathrin-mediated endocytosis signalling | Hepatic Fibrosis / Hepatic Stellate Cell Activation | Acute Phase Response Signaling | Type I Diabetes Mellitus Signaling |
| 7 | Extrinsic prothrombin activation pathway | Reelin Signaling in Neurons | Activation of IRF by Cytosolic Pattern Recognition Receptors | Agranulocyte Adhesion and Diapedesis |
| 8 | PXR/RXR Activation | Regulation of the Epithelial-Mesenchymal Transition Pathway | LXR/RXR Activation | IL-10 Signaling |
| 9 | Atherosclerosis signalling | Agrin Interactions at Neuromuscular Junction | Role of Macrophages, Fibroblasts and Endothelial Cells in Rheumatoid Arthritis | Role of Macrophages, Fibroblasts and Endothelial Cells in Rheumatoid Arthritis |
| 10 | TR/RXR activation | STAT3 Pathway | IL-6 Signaling | Glucocorticoid Receptor Signaling |
|  | **Top pathways associated with down-regulated genes in *F. tularensis* infection** | | | |
|  | **24 h p.i.** | **48 h p.i.** | **72 h p.i.** | **96 h p.i.** |
|  | EIF2 Signaling | EIF2 Signaling | Oxidative Phosphorylation | Axonal Guidance Signaling |
|  | Regulation of eIF4 and p70S6K Signaling | Oxidative Phosphorylation | Mitochondrial Dysfunction | Hepatic Fibrosis / Hepatic Stellate Cell Activation |
|  | mTOR Signaling | Mitochondrial Dysfunction | EIF2 Signaling | ILK Signaling |
|  | VEGF Signaling | Regulation of eIF4 and p70S6K Signaling | Calcium Signaling | Nitric Oxide Signaling in the Cardiovascular System |
|  | Systemic Lupus Erythematosus Signaling | mTOR Signaling | Regulation of eIF4 and p70S6K Signaling | Inhibition of Angiogenesis by TSP1 |
|  | Protein Kinase A Signaling | Protein Ubiquitination Pathway | Cellular Effects of Sildenafil (Viagra) | LPS/IL-1 Mediated Inhibition of RXR Function |
|  | Integrin Signaling | Calcium Signaling | mTOR Signaling | Tight Junction Signaling |
|  | Protein Ubiquitination Pathway | Agranulocyte Adhesion and Diapedesis | LPS/IL-1 Mediated Inhibition of RXR Function | Agranulocyte Adhesion and Diapedesis |
|  | Calcium Signaling | NRF2-mediated Oxidative Stress Response | Protein Kinase A Signaling | eNOS Signaling |
|  | Agrin Interactions at Neuromuscular Junction | Systemic Lupus Erythematosus Signaling | Phospholipase C Signaling | Xenobiotic Metabolism Signaling |

**Table S4.** The most statistically significant up and down regulated pathways across the time course of infection in the mouse lung infected with *Y. pestis* by the aerosol route.

|  | **Top pathways associated with up-regulated genes in *Y. pestis* infection** | | |
| --- | --- | --- | --- |
|  | **24 h p.i.** | **48 h p.i.** | **72 h p.i.** |
| 1 | Epithelial Adherens Junction Signaling | Hepatic Fibrosis / Hepatic Stellate Cell Activation | Granulocyte Adhesion and Diapedesis |
| 2 | Hepatic Fibrosis / Hepatic Stellate Cell Activation | Agranulocyte Adhesion and Diapedesis | IL-10 Signaling |
| 3 | Glucocorticoid Receptor Signaling | Granulocyte Adhesion and Diapedesis | IL-6 Signaling |
| 4 | Factors Promoting Cardiogenesis in Vertebrates | IL-10 Signaling | Agranulocyte Adhesion and Diapedesis |
| 5 | Axonal Guidance Signaling | Glucocorticoid Receptor Signaling | Acute Phase Response Signaling |
| 6 | Neuroprotective Role of THOP1 in Alzheimer's Disease | Activation of IRF by Cytosolic Pattern Recognition Receptors | Role of Macrophages, Fibroblasts and Endothelial Cells in Rheumatoid Arthritis |
| 7 | Cellular Effects of Sildenafil (Viagra) | Role of Macrophages, Fibroblasts and Endothelial Cells in Rheumatoid Arthritis | Glucocorticoid Receptor Signaling |
| 8 | p53 Signaling | PPAR Signaling | Hepatic Fibrosis / Hepatic Stellate Cell Activation |
| 9 | VEGF Signaling | IL-6 Signaling | Death Receptor Signaling |
| 10 | Actin Cytoskeleton Signaling | TREM1 Signaling | Dendritic Cell Maturation |
|  | **Top pathways associated with down-regulated genes in *Y. pestis* infection** | | |
|  | **24 h p.i.** | **48 h p.i.** | **72 h p.i.** |
| 1 | Oxidative Phosphorylation | EIF2 Signaling | Axonal Guidance Signaling |
| 2 | Mitochondrial Dysfunction | Regulation of eIF4 and p70S6K Signaling | EIF2 Signaling |
| 3 | EIF2 Signaling | mTOR Signaling | Glutathione-mediated Detoxification |
| 4 | Regulation of eIF4 and p70S6K Signaling | Xenobiotic Metabolism Signaling | Human Embryonic Stem Cell Pluripotency |
| 5 | mTOR Signaling | NRF2-mediated Oxidative Stress Response | Oxidative Phosphorylation |
| 6 | Glutathione-mediated Detoxification | Oxidative Phosphorylation | mTOR Signaling |
| 7 | TCA Cycle II (Eukaryotic) | Glioma Invasiveness Signaling | Regulation of the Epithelial-Mesenchymal Transition Pathway |
| 8 | DNA Methylation and Transcriptional Repression Signaling | B Cell Development | Basal Cell Carcinoma Signaling |
| 9 | Protein Ubiquitination Pathway | Mitochondrial Dysfunction | Xenobiotic Metabolism Signaling |
| 10 | Pyrimidine Deoxyribonucleotides De Novo Biosynthesis I | Antiproliferative Role of Somatostatin Receptor 2 | Regulation of eIF4 and p70S6K Signaling |

**Table S5.** Complete list of statistically significant up- or downregulated transcripts within all three bacterial infections (supplied as a separate file).
